# Plasma endostatin at intensive care admission is independently associated with acute kidney injury, dialysis, and mortality in COVID-19

**DOI:** 10.1186/s40635-025-00748-6

**Published:** 2025-04-03

**Authors:** Hazem Koozi, Jonas Engström, Ahmad Zwawi, Martin Spångfors, Ingrid Didriksson, Anders Larsson, Hans Friberg, Attila Frigyesi

**Affiliations:** 1 Department of Clinical Sciences, Anaesthesiology and Intensive Care, Lund University, 22185 Lund, Sweden; 2 Department of Anaesthesia and Intensive Care, Kristianstad Hospital, 29133 Kristianstad, Sweden; 3 Faculty of Medicine, Lund University, 22184 Lund, Sweden; 4 Department of Intensive and Perioperative Care, Skåne University Hospital, 20502 Malmö, Sweden; 5 Department of Medical Sciences, Clinical Chemistry, Uppsala University, 75185 Uppsala, Sweden; 6 Department of Intensive and Perioperative Care, Skåne University Hospital, 22185 Lund, Sweden

**Keywords:** COVID-19, Intensive care, Critical care, Endostatin, Acute kidney injury, Renal replacement therapy, Mortality

## Abstract

**Background:**

Critical COVID-19 is associated with high mortality, and acute kidney injury (AKI) is common. Endostatin has emerged as a promising prognostic biomarker for predicting AKI and mortality in intensive care. This study aimed to investigate plasma endostatin at intensive care unit (ICU) admission as a biomarker for AKI, renal replacement therapy (RRT), and 90-day mortality in COVID-19.

**Methods:**

A pre-planned retrospective analysis of a prospectively collected cohort of admissions with a primary SARS-CoV-2 infection to six ICUs in southern Sweden between May 2020 and May 2021 was undertaken. Endostatin at ICU admission was evaluated with multivariable logistic regression analyses adjusted for age, sex, C-reactive protein, and creatinine. Net reclassification index analyses were also performed.

**Results:**

Four hundred eighty-four patients were included. Endostatin showed a non-linear association with AKI, RRT, and 90-day mortality. Endostatin levels of 100–200 ng/mL were associated with AKI on ICU day 1 (OR 5.1, 95% CI 1.5–18,* p* = 0.0097), RRT during the ICU stay (OR 3.5, 95% CI 1.1–12,* p* = 0.039), and 90-day mortality (OR 4.2, 95% CI 1.6–11,* p* = 0.0037). Adding endostatin to creatinine improved prediction of AKI on ICU day 1, while adding it to a model containing age, sex, CRP, and creatinine improved prediction of both AKI on ICU day 1 and 90-day mortality, but not RRT.

**Conclusions:**

Endostatin at ICU admission was independently associated with AKI, RRT, and 90-day mortality in ICU patients with COVID-19. In addition, endostatin improved the prediction of AKI and 90-day mortality, highlighting its potential as a biomarker for early risk stratification in intensive care.

## Background

Critical COVID-19 is associated with high mortality [1]. Approximately 10% of hospitalised COVID-19 patients develop acute kidney injury (AKI), a rate that increases to 26% among those admitted to the intensive care unit (ICU) [2]. Renal replacement therapy (RRT) is frequently required in critically ill COVID-19 patients, with rates around 20%, and a significant proportion remaining dependent on RRT at discharge [3].

Established indicators of renal function, such as serum creatinine and urine output, are unreliable, typically identifying AKI during its later stages when renal filtration is already significantly impaired. Numerous biomarkers have been studied to improve the detection of AKI at earlier stages. Early detection of AKI using more specific biomarkers could improve diagnostic accuracy and enhance patient outcomes by allowing timely recognition and intervention [4].

Endostatin is a fragment of collagen XVIII derived from the basement membrane. It is expressed in the kidneys and other tissues [5, 6]. Increased plasma levels of endostatin have been observed in cardiovascular, pulmonary, and renal diseases [7–11]. Endostatin has been associated with disease severity in critical COVID-19 in a small study and has shown potential as a biomarker for AKI and mortality in general ICU cohorts [12–15].

### Objectives

This study aimed to investigate plasma endostatin on ICU admission as a biomarker for AKI, RRT, and mortality in critical COVID-19.

## Methods

### Study design and setting

A pre-planned retrospective analysis of a prospectively collected multicenter cohort was conducted as part of the Swecrit project [16]. The study involved six ICUs: two general, one cardiothoracic, and one infectious disease ICU at Skåne University Hospital (Lund and Malmö, Sweden), as well as two general ICUs at Helsingborg Hospital and Kristianstad Hospital in Sweden. ICU admissions were consecutively included between May 11, 2020, and May 10, 2021, with follow-up monitoring extending to at least 101 days post-ICU admission.

### Participants

Adult patients with laboratory-confirmed SARS-CoV-2 infection were eligible for inclusion. Exclusion criteria were ICU admission primarily due to reasons other than COVID-19, lack of consent, and missing endostatin on ICU admission.

### Data sources and variables

Data were obtained from the Swecrit-COVID-IR database, as detailed by Didriksson et al. [17]. Blood samples were collected upon ICU admission, on ICU days 2 and 7, and at the 3- and 12-month follow-up visits.

Endostatin, creatinine, C-reactive protein (CRP), neutrophil gelatinase-associated lipocalin (NGAL), and calprotectin were retrospectively batch-analysed from these blood samples. All other laboratory values were automatically extracted from the electronic medical records.

Endostatin was analysed in blood sampled in ethylenediamine tetraacetic acid (EDTA) vacutainers and centrifuged to obtain EDTA plasma. The plasma samples were aliquoted and stored in the Swecrit biobank at $-80^{\circ }$C. Samples had to be collected within 6 h of ICU admission. In cases where the sampling time was missing, samples were included if the freezing time fell within the 6-h time frame.

Endostatin analyses were performed using commercial sandwich kits (DY1098, R&D Systems, Minneapolis, MN, USA). A monoclonal antibody specific for endostatin was coated onto microtiter plates. The plates were blocked with bovine serum albumin and then washed, and samples and standards were added to the wells, after which the peptide was bound to the immobilised antibodies. A biotinylated endostatin-specific antibody was added after washing. A streptavidin–HR conjugate was added to the wells after incubation and washing, and a substrate solution was added after another incubation and washing cycle. The absorbance was measured in a SpectraMax 250 (Molecular Devices, Sunnyvale, CA, USA). Endostatin values were determined by comparing the optical density of the samples with the standard curve. All assays were calibrated against highly purified recombinant human endostatin. Measurements were performed blinded, without knowledge of the clinical diagnoses. The total coefficient of variation for the endostatin assay was approximately 6%. All blood samples were subjected to the same degree of dilution. Routine quality control procedures were performed to detect any temporal variations in endostatin measurements. In addition, all samples were assessed for hemolysis, and any occurrence was documented.

Confirmation of SARS-CoV-2 infection relied on real-time reverse transcriptase–polymerase chain reaction (RT–PCR) testing conducted on nasal or pharyngeal swabs or lower respiratory airway aspirates.

Simplified Acute Physiology Score 3 (SAPS-3) and Sequential Organ Failure Assessment (SOFA) parameters, survival data, age, and sex were automatically extracted from the Patient Administrative System for Intensive Care Units (PASIVA). PASIVA is a digital system where treating clinicians submit data to the Swedish Intensive Care Registry (SIR) during patients’ ICU stay. PASIVA is linked to the national Swedish population register. Data on comorbidities and treatments was collected manually from electronic medical records.

AKI was defined according to Kidney Disease Improving Global Outcomes (KDIGO) guidelines [18].

ICU day 1 was defined as starting at 6 am on the morning following ICU admission.

### Study size

The sample size was determined by the number of ICU admissions with COVID-19 during the study period and patient consent.

### Bias

Treating clinicians did not know the endostatin levels. Trained data collectors performed the manual data recording. Guidelines for data collection were standardised.

### Statistical analysis

All statistical analyses were performed in R [19].

Admission characteristics, laboratory values, and outcomes were summarised as medians with interquartile ranges, except for the Charlson Comorbidity Index (CCI), Clinical Frailty Score, SOFA score, and Glasgow Coma Scale, which were summarised as means with standard deviations due to their distribution. Differences between groups were evaluated using the Chi-square test of Independence, Fisher’s Exact Test, or Analysis of Variance as appropriate.

Local polynomial regression was employed to show trends in the association between endostatin, outcomes, and creatinine [20].

Multivariable logistic regressions were conducted to assess the independent association of endostatin with AKI on ICU day 1, RRT during the ICU stay, and 90-day mortality. Patients with AKI at ICU admission were excluded from analyses for AKI on ICU day 1. Endostatin was analysed as a categorical variable for logistic regression analyses and net reclassification improvement (NRI). Values < 50 ng/mL represented a baseline state, as supported by studies reporting similar endostatin levels in healthy individuals and a mixed elderly population [10, 21]. A cutoff of 100 ng/mL was chosen based on evidence linking endostatin levels around 100 ng/mL to increased mortality in critically ill patients with AKI, as well as for pragmatic reasons [14]. Further categorisation of endostatin levels was based on the distribution of endostatin levels. The models were adjusted for age, sex, CRP at ICU admission, and creatinine at ICU admission. CRP and creatinine underwent log transformation and subsequent z-normalisation before regression analyses to facilitate the calculation and comparisons of odds ratios (ORs). The results were presented as adjusted ORs with 95% confidence intervals (CI). NRI was calculated to assess the added predictive value of endostatin. Predicted probabilities from each model were categorised into risk strata based on the tertiles of the overall probability distribution.

A *p*-value of less than 0.05 was considered statistically significant.

## Results

### Participants

**Fig. 1:**
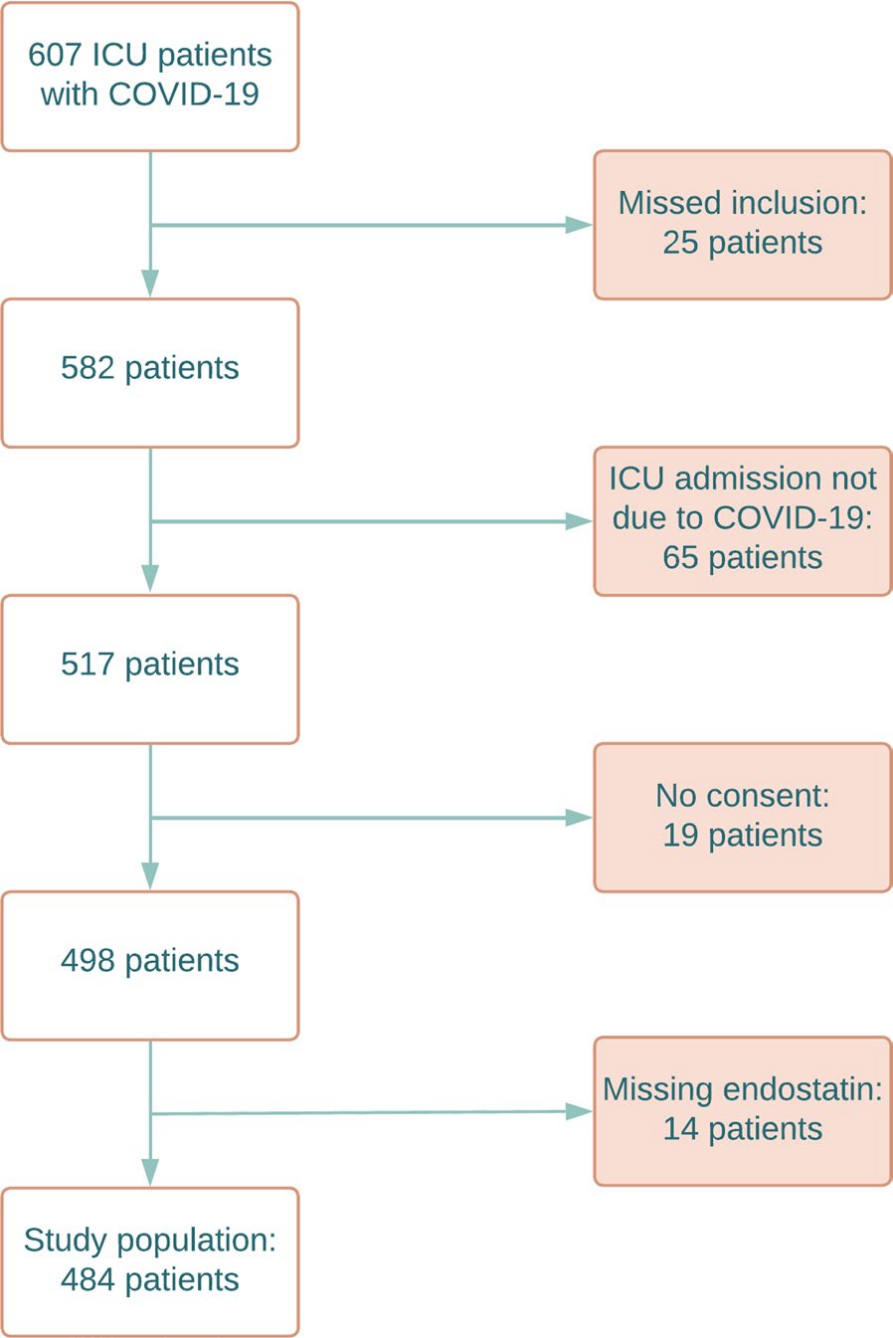
Flow chart of included and excluded patients. *ICU* Intensive Care Unit

During the study period, 607 patients were admitted to the ICU with COVID-19. Of these, 25 missed inclusion, 65 were admitted for reasons other than COVID-19, 19 lacked consent, and 14 patients had missing admission endostatin. This left a final study population of 484 patients, see Fig. 1.

### Descriptive data

**Fig. 2:**
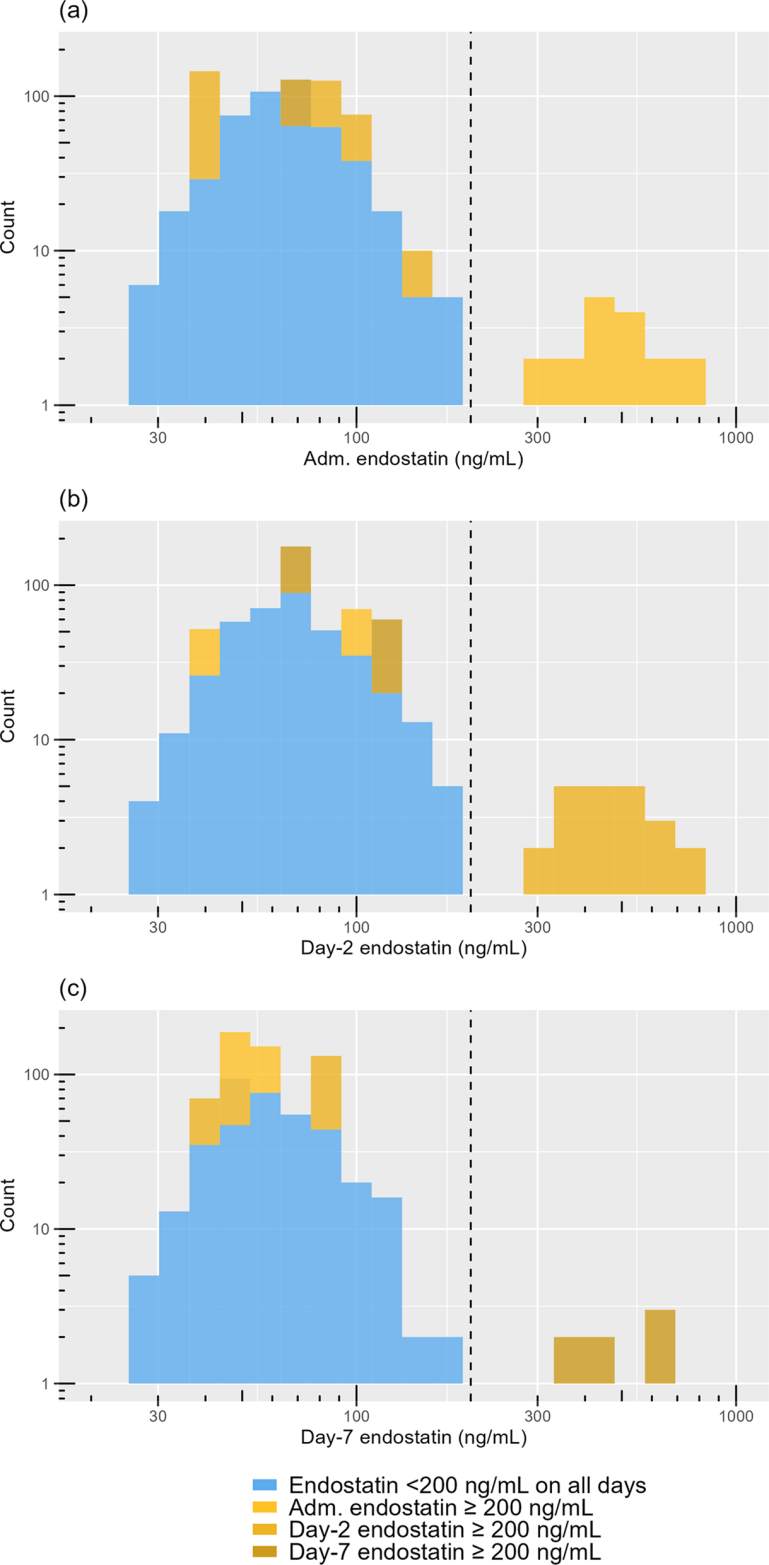
Histograms of plasma endostatin levels measured at ICU admission (**a**), on ICU day 2 (**b**), and on ICU day 7 (**c**). Patients are colour-coded based on the earliest day they reached endostatin > 200 ng/mL: light yellow for ICU admission, medium yellow for ICU day 2, and dark yellow for ICU day 7. Patients with endostatin levels consistently below 200 ng/mL are shown in blue. The dashed vertical line indicates the 200 ng/mL threshold. The axes have logarithmic scales. *Adm* Admission, *ICU* Intensive Care Unit

The median age was 66 years (interquartile range [IQR] 56–73 years). Males constituted 74% of the study population. The mean CCI was 2.9 (standard deviation [SD] 1.9). The median SAPS-3 score was 60 (IQR 50–69). The mean SOFA score was 7.4 (SD 3.2) at ICU admission. The prevalence of chronic kidney disease (CKD) was 4.0%. Median endostatin on ICU admission was 61 ng/mL (IQR 50–83), see Fig. 2 for a histogram. The median CRP at ICU admission was 150 (IQR 83–230). The median creatinine at ICU admission was 77 (IQR 65–100). The proportion of patients in need of invasive mechanical ventilation was 72%.

Characteristics and categorisation of the study population based on admission endostatin levels are presented in Table 1. The group with endostatin 100–200 ng/mL had the highest rates of beta-blocker treatment, complicated diabetes mellitus, CKD, CCI, Clinical Frailty Scale, SAPS-3 score, and SOFA score. This group also had the highest levels of creatinine, NGAL, and calprotectin. Aspirin and statin treatment rates and interleukin-6 levels increased with higher endostatin.

### Outcomes

AKI during the ICU stay occurred in 61% (*n* = 295), with 36% (*n* = 105) of cases present on ICU admission and 25% (*n* = 73) developing on ICU day 1. AKI status during the ICU stay was missing for 15% (*n* = 72) of the population. A total of 13% (*n* = 65) of the study population received RRT. Ninety-day mortality was 39% (*n* = 187). The rates of AKI, RRT, and mortality were highest in the endostatin 100–200 ng/mL group, see Table 1.

**Table 1:**
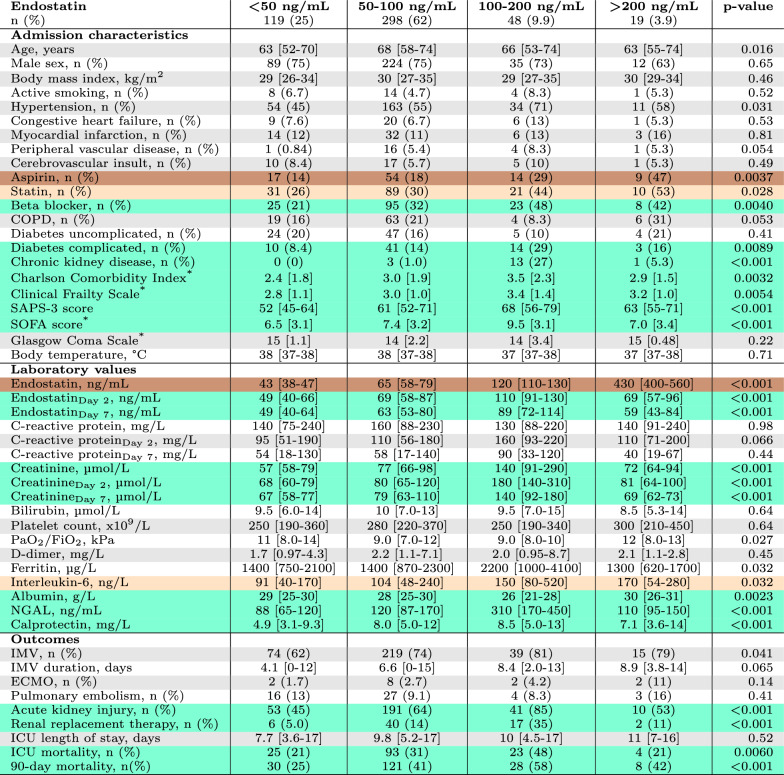
Characteristics of the study population based on plasma endostatin at ICU admission

Laboratory values are at ICU admission unless otherwise specified. Values are presented as medians with interquartile ranges unless otherwise specified. *P*-values are calculated using the appropriate Chi-square test of Independence or Fisher’s Exact Test. Torqouise indicates *p* < 0.01 with the endostatin 100–200 ng/mL group having the highest rate or value. Light brown indicates *p* < 0.05 with the endostatin > 200 ng/ml group having the highest rate or value. Dark brown indicates *p* < 0.01 with the endostatin > 200 ng/mL group having the highest rate or value

*COPD* Chronic Obstructive Pulmonary Disease, *SAPS-3* Simplified Acute Physiology Score 3, *SOFA* Sequential Organ Failure Assessment, *PaO*_2_ Arterial Partial Pressure of Oxygen, *FiO*_2_ Fraction of Inspired Oxygen, *NGAL* Neutrophil Gelatinase-Associated Lipocalin, *IMV* Invasive Mechanical Ventilation, *ECMO* Extracorporeal Membrane Oxygenation, *ICU* Intensive Care Unit

^*^Presented as means with standard deviations, and *p*-value calculated with Analysis of Variance

### Relationship with outcomes and creatinine

**Fig. 3:**
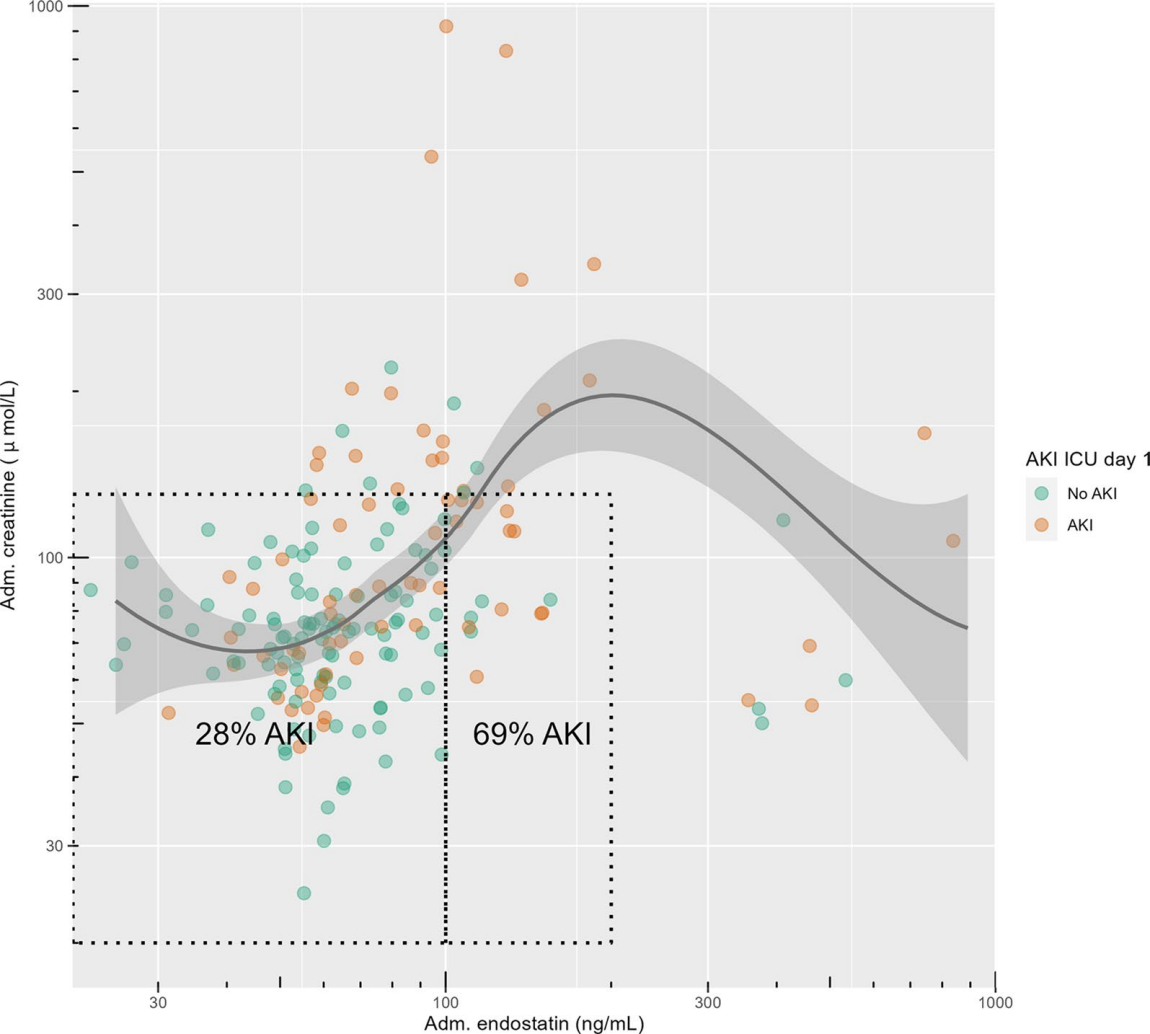
Relationship between plasma endostatin at ICU admission, plasma creatinine at ICU admission, and AKI on ICU day 1 visualised in a scatter plot. The box to the left indicates patients with creatinine 25–130 $\upmu$mol/L and endostatin 20–100 ng/mL, while the right box indicates creatinine 25–130 $\upmu$mol/L and endostatin 100–200 ng/mL. *p* = 0.0038 for the AKI rate comparison between the two boxes.* P*-value calculated using Fisher’s exact test. The axes have logarithmic scales. Continuous lines show smoothed conditional means, while the accompanying shading indicates the corresponding 95% confidence interval. *Adm* Admission, *AKI* Acute Kidney Injury, *ICU* Intensive Care Unit

**Fig. 4:**
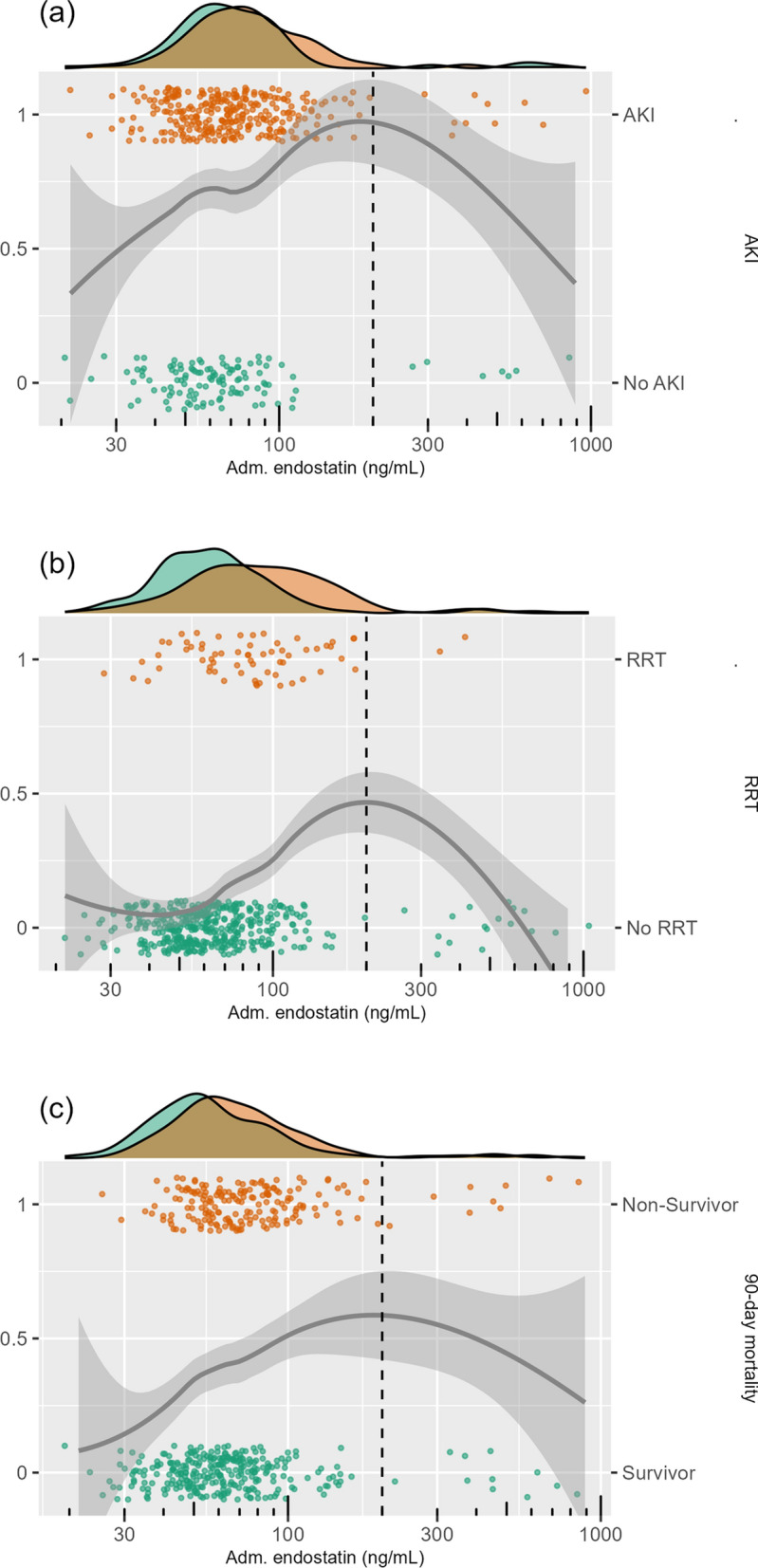
Relationship between plasma endostatin at ICU admission and AKI, RRT, and 90-day mortality visualised in scatter and density plots. The x-axes have logarithmic scales. The points show the outcome (AKI, RRT, or 90-day mortality) versus plasma endostatin level in individual patients. Continuous lines represent smoothed conditional means, while the accompanying shading indicates the corresponding 95% confidence intervals. *Adm* Admission, *AKI* Acute Kidney Injury, *RRT* Renal Replacement Therapy

The relationships between endostatin, creatinine, AKI, RRT, and mortality are visualised in Figs. 3 and 4.

### Main results

#### AKI

Logistic regression analysis (*n* = 307), adjusted for age, sex, CRP, and creatinine, showed that endostatin 100–200 ng/mL was associated with AKI on ICU day 1 (OR 5.1, 95% CI 1.5–18,* p* = 0.0097), see Table 2. Adding endostatin to creatinine resulted in an NRI of 0.14 (95% CI 0.026$-$0.24) for predicting AKI on ICU day 1. Adding endostatin to a model containing age, sex, CRP, and creatinine resulted in an NRI of 0.13 (95% CI 0.010$-$0.23).

**Table 2:** Association between plasma endostatin at ICU admission and the risk of AKI on ICU day 1, RRT during ICU stay, and 90-day mortality, adjusted for age, sex, C-reactive protein, and creatinine, using multivariable logistic regression

Variable	AKI		RRT		90-day mortality	
	OR (95% CI)	*p*-value	OR (95% CI)	*p*-value	OR (95% CI)	*p*-value
Endostatin (ng/mL)						
< 50	1.0 (reference)	ns	1.0 (reference)	ns	1.0 (reference)	ns
50–100	1.5 (0.65–3.6)	0.38	2.4 (1.0–6.5)	0.060	1.6 (0.93–2.7)	0.096
100–200	5.1 (1.5–18)	0.0097	3.5 (1.1–12)	0.039	4.2 (1.6–11)	0.0037
> 200	2.5 (0.63–9.5)	0.18	2.1 (0.29–10)	0.38	2.2 (0.69–6.8)	0.18
Age	1.0 (0.99–1.0)	0.30	1.0 (0.98–1.0)	0.95	1.1 (1.1–1.1)	< 0.001
Male sex	0.67 (0.34–1.4)	0.26	1.3 (0.64–2.6)	0.53	0.81 (0.49–1.3)	0.41
C-reactive protein	1.2 (0.84–1.7)	0.38	0.84 (0.65–1.1)	0.18	0.86 (0.68–1.1)	0.17
Creatinine	2.8 (1.8–4.6)	< 0.001	1.8 (1.4–2.5)	< 0.001	1.1 (0.84–1.4)	0.57

*AKI* Acute Kidney Injury, *RRT* Renal Replacement Therapy, *OR* Odds Ratio, *CI* Confidence Interval

#### RRT

Logistic regression analysis (*n* = 483), adjusted for age, sex, CRP, and creatinine, showed that endostatin 100–200 ng/mL was associated with RRT during the ICU stay (OR 3.5, 95% CI 1.1–12, *p* = 0.039), see Table 2. Adding endostatin to creatinine resulted in an NRI of $-$0.017 (95% CI $-$0.18 to 0.15) for predicting RRT during the ICU stay. Adding endostatin to a model containing age, sex, CRP, and creatinine resulted in an NRI of 0.087 (95% CI $-$0.063 to 0.24).

#### Mortality

Logistic regression analysis (*n* = 483), adjusted for age, sex, CRP, and creatinine, showed that endostatin 100–200 ng/mL was associated with 90-day mortality (OR 4.2, 95% CI 1.6–11, *p* = 0.0037), see Table 2. Adding endostatin to a model containing age, sex, CRP, and creatinine resulted in an NRI of 0.076 (95% CI 0.016$-$0.13) for predicting 90-day mortality.

## Discussion

In this study of critically ill COVID-19 patients, plasma endostatin levels of 100–200 ng/mL at ICU admission were associated with AKI development on ICU day 1, RRT need, and 90-day mortality independent of age, sex, CRP, and creatinine. Adding endostatin to creatinine improved AKI prediction on ICU day 1 but not RRT. Incorporating endostatin into a model containing age, sex, CRP, and creatinine enhanced the prediction of both AKI on ICU day 1 and 90-day mortality, but not RRT.

The relationship between endostatin and AKI, RRT, and 90-day mortality was non-linear, with the highest risk observed near 200 ng/mL. SAPS-3, SOFA score, lactate, AKI, RRT, and mortality were highest with endostatin levels of 100–200 ng/mL, suggesting a correlation with organ dysfunction. Furthermore, this group had the highest CCI and prevalence of diabetes and CKD. The strong association between endostatin and these markers of disease severity supports the role of endothelial dysfunction and dysregulated angiogenesis in the pathophysiology of critical COVID-19. Approximately 4% of the population had very high endostatin levels (> 200 ng/mL), a finding not previously reported in critically ill patients, with or without COVID-19 [12, 13, 22]. The reason for the decreasing AKI, RRT, and mortality rate with endostatin > 200 ng/mL is not entirely clear. We hypothesised that the high rate of aspirin treatment (47%) in the endostatin > 200 ng/mL group may have provided protective effects against AKI. Endostatin has been associated with platelet activation in COVID-19, and platelet activation has been implicated in the pathophysiology of COVID-19-associated AKI [23, 24]. Furthermore, aspirin use has been linked to reduced mortality in COVID-19 in a large meta-analysis [25]. Another possible explanation is that the effects of endostatin follow a non-linear, concentration-dependent pattern. This has been observed in oncological research, both in vitro and in vivo, where endostatin inhibits endothelial cell proliferation and migration in a dose-dependent manner up to a certain threshold. Beyond this point, however, further increases in endostatin levels paradoxically decrease its inhibitory effects, indicating a biphasic or U-shaped response. Such dose–response relationships have also been shown for other angiogenesis inhibitors [26, 27].

In a general ICU setting, a 2016 study showed that adding endostatin to a predictive model enhanced AKI prediction [13]. Similarly, a recent study concluded that admission endostatin, age, and creatinine effectively predicted AKI and RRT [15]. In contrast, a larger multicenter study reported limited utility for endostatin as a predictive marker for AKI and RRT, despite levels increasing with KDIGO stages [22]. Unlike this study, this investigation was not focused on COVID-19 patients, had a lower AKI rate, and did not stratify patients by endostatin levels. The low area under the curve of endostatin observed in that study could reflect a non-linear relationship between endostatin, AKI, and RRT, as highlighted in the present study.

Endostatin has also been linked to mortality in critically ill patients with AKI and in a general ICU cohort [14, 15]. In critical COVID-19, elevated endostatin levels have been associated with hypoxia and mortality [12]. These findings further underscore the prognostic value of endostatin in critical disease.

Endostatin is expressed in various tissues, including the renal tubular epithelium, Bowman’s capsule, and the basement membrane of the kidneys and lungs [28]. Therefore, it is plausible that endostatin may be released during pulmonary and renal injury. With a molecular weight of 20 kDa, endostatin is expected to be filtered through the glomeruli [29, 30]. Declining GFR during AKI likely contributes to elevated plasma levels [31]. In addition, experimental data suggest that endostatin may play an active role in AKI development [32–34]. Thus, elevated endostatin levels may reflect kidney injury and reduced GFR and actively contribute to AKI.

Renal impairment is often detected late, as creatinine changes appear only after significant kidney injury [35, 36]. Incorporating novel biomarkers like endostatin could enhance early risk stratification and AKI diagnosis, allowing timely intervention for reversible factors like hypoperfusion and nephrotoxic exposures, ultimately improving ICU outcomes.

The observed association between endostatin, RRT, and mortality may partly be explained by its strong relationship with AKI. Given that AKI is a well-established risk factor for RRT and mortality, it is possible that endostatin primarily reflects AKI severity rather than being independently associated with RRT and mortality per se. Even though factors beyond AKI classification influence RRT need, it is often a consequence of severe AKI, making it difficult to distinguish between the two fully. However, it may be more clinically relevant to interpret RRT as a severity marker of AKI rather than as an isolated outcome. While endostatin was associated with RRT, its lack of predictive improvement likely reflects the complexity of RRT need, the strong predictive role of creatinine, and the potential need for dynamic biomarker assessment rather than a single admission value. RRT initiation is not solely determined by AKI severity but also by fluid balance, acid–base status, and haemodynamic factors, which may limit endostatin’s added value in RRT prediction. These findings suggest that while endostatin enhances AKI risk stratification, its role in guiding RRT prediction may be more limited.

Future research should include larger studies to validate these findings and explore the intriguing observation of decreasing risk of poor outcomes with very high endostatin levels (> 200 ng/mL). In addition, larger studies in other ICU cohorts beyond COVID-19 are needed to assess endostatin’s broader applicability. Combining endostatin with other emerging AKI biomarkers could enhance diagnostic accuracy and risk stratification, potentially guiding therapeutic interventions in future clinical trials. Further research is also needed to clarify the precise role of endostatin in AKI development and its potential as a therapeutic target.

The multicenter design of this study enhances its generalisability to critically ill COVID-19 patients. Its prospective approach enabled systematic follow-up and detailed data collection on comorbidities, laboratory parameters, and daily AKI status. Robust statistical models, adjusted for age, sex, CRP, and creatinine, minimised confounding and reinforced the associations between endostatin levels, AKI, RRT, and 90-day mortality.

The observational design introduces the possibility of residual confounding despite adjustments for age, sex, CRP, and creatinine. Factors such as ICU burden and SARS-CoV-2 variants may have influenced patient outcomes during the study period. While we did not have variant-specific data for individual patients, surveillance data indicate that the B.1.1.7 (alpha) variant was predominant in Sweden at the time [37]. Although the ICU burden has been associated with increased mortality in critically ill COVID-19 patients, we believe this is unlikely to have significantly impacted our findings, as our models adjusted for key confounders, and ICU admission policies remained relatively consistent across centres [17]. Although the study involved multiple ICUs in Southern Sweden, the findings may not be fully generalisable to other regions or healthcare settings. The study’s focus on COVID-19 also limits its applicability to non-COVID ICU populations, as mechanisms of AKI may differ. The non-linear relationship between endostatin and outcomes may add complexity to its interpretation. Endostatin levels were categorised based on a combination of prior evidence, the observed distribution in our cohort, and pragmatic considerations. However, we acknowledge that endostatin is a continuous variable, and future studies should explore optimal thresholds and alternative modelling approaches. Furthermore, the subgroup with very high endostatin levels (> 200 ng/mL) was relatively small, potentially limiting statistical power to draw firm conclusions. This prevented a meaningful sensitivity analysis of aspirin’s potential protective effects in this group. Larger studies are needed to characterise patients with very high endostatin levels better.

## Conclusions

This multicentre study demonstrates that plasma endostatin at ICU admission was independently associated with AKI, RRT need, and 90-day mortality in critically ill COVID-19 patients after adjusting for age, sex, CRP and creatinine. A non-linear relationship was observed, with the highest risks at levels between 100–200 ng/mL. Endostatin improved prediction of AKI and 90-day mortality, highlighting its potential as a valuable biomarker for early risk stratification in intensive care, particularly in COVID-19.

## Data Availability

The data sets generated and analysed during the current study are not publicly available due to limitations in the ethical approval of the study and data management policies of Region Skåne. However, they are available from the corresponding author on request.

## Abbreviations

AKI: Acute kidney injury
CCI: Charlson Comorbidity Index
CI: Confidence interval
CKD: Chronic kidney disease
CRP: C-reactive protein
EDTA: Ethylenediamine tetraacetic acid
GFR: Glomerular filtration rate
ICU: Intensive Care Unit
IQR: Interquartile range
KDIGO: Kidney Disease Improving Global Outcomes
NGAL: Neutrophil gelatinase-associated lipocalin
NRI: Net reclassification improvement
OR: Odds ratio
PASIVA: Patient Administrative System for Intensive Care Units
RRT: Renal Replacement Therapy
RT–PCR: Real-time Reverse Transcriptase–Polymerase Chain Reaction
SAPS-3: Simplified Acute Physiology Score 3
SIR: Swedish Intensive Care Registry
SOFA: Sequential Organ Failure Assessment
STROBE: Strengthening the Reporting of Observational Studies in Epidemiology
